# Combining public datasets for automated tooth assessment in panoramic radiographs

**DOI:** 10.1186/s12903-024-04129-5

**Published:** 2024-03-26

**Authors:** Niels van Nistelrooij, Khalid El Ghoul, Tong Xi, Anindo Saha, Steven Kempers, Max Cenci, Bas Loomans, Tabea Flügge, Bram van Ginneken, Shankeeth Vinayahalingam

**Affiliations:** 1 Department of Oral and Maxillofacial Surgery, Radboud University Nijmegen Medical Centre, Postal Number 590, P.O. Box 9101, Nijmegen, 6500 HB The Netherlands; 2 Department of Radiology, Radboud University Medical Center, Geert Grooteplein Zuid 10, Nijmegen, 6525 GA The Netherlands; 3 Department of Oral and Maxillofacial Surgery, Charité - Universitätsmedizin Berlin, Corporate Member of Freie Universität Berlin and Humboldt-Universität zu Berlin, Hindenburgdamm 30, 12203 Berlin, Germany; 4 Department of Oral and Maxillofacial Surgery, Erasmus MC, Dr. Molewaterplein 40, Rotterdam, The Netherlands; 5 Einstein Center for Digital Future, Wilhelmstraße 67, Berlin, Germany; 6 Department of Dentistry, Radboud University Medical Center, Ph. Van Leydenlaan 25, Nijmegen, 6525 EX The Netherlands

**Keywords:** Panoramic radiograph, Artificial intelligence, Public datasets, Tooth segmentation, Diagnostic classification

## Abstract

**Objective:**

Panoramic radiographs (PRs) provide a comprehensive view of the oral and maxillofacial region and are used routinely to assess dental and osseous pathologies. Artificial intelligence (AI) can be used to improve the diagnostic accuracy of PRs compared to bitewings and periapical radiographs. This study aimed to evaluate the advantages and challenges of using publicly available datasets in dental AI research, focusing on solving the novel task of predicting tooth segmentations, FDI numbers, and tooth diagnoses, simultaneously.

**Materials and methods:**

Datasets from the OdontoAI platform (tooth instance segmentations) and the DENTEX challenge (tooth bounding boxes with associated diagnoses) were combined to develop a two-stage AI model. The first stage implemented tooth instance segmentation with FDI numbering and extracted regions of interest around each tooth segmentation, whereafter the second stage implemented multi-label classification to detect dental caries, impacted teeth, and periapical lesions in PRs. The performance of the automated tooth segmentation algorithm was evaluated using a free-response receiver-operating-characteristics (FROC) curve and mean average precision (mAP) metrics. The diagnostic accuracy of detection and classification of dental pathology was evaluated with ROC curves and F1 and AUC metrics.

**Results:**

The two-stage AI model achieved high accuracy in tooth segmentations with a FROC score of 0.988 and a mAP of 0.848. High accuracy was also achieved in the diagnostic classification of impacted teeth (F1 = 0.901, AUC = 0.996), whereas moderate accuracy was achieved in the diagnostic classification of deep caries (F1 = 0.683, AUC = 0.960), early caries (F1 = 0.662, AUC = 0.881), and periapical lesions (F1 = 0.603, AUC = 0.974). The model’s performance correlated positively with the quality of annotations in the used public datasets. Selected samples from the DENTEX dataset revealed cases of missing (false-negative) and incorrect (false-positive) diagnoses, which negatively influenced the performance of the AI model.

**Conclusions:**

The use and pooling of public datasets in dental AI research can significantly accelerate the development of new AI models and enable fast exploration of novel tasks. However, standardized quality assurance is essential before using the datasets to ensure reliable outcomes and limit potential biases.

## Introduction

Panoramic radiographs (PRs) provide a two-dimensional (2D) radiographic view of the upper and lower jaw, including the teeth and adjacent osseous structures. PRs are commonly used in dentistry and oral and maxillofacial surgery for diagnostic purposes due to their easy acquisition, limited radiation exposure, and comprehensive field of view. Although frequently used for assessing tooth impaction and identifying cysts, tumors, and other bony or osteolytic pathologies, the diagnostic accuracy of PRs is limited by interobserver variability and the 2D representation of complex 3D maxillofacial structures [1–3].

Pathologies of odontogenic origin, such as caries, periapical lesions, and impacted teeth, are routinely diagnosed through clinical and radiographic assessment [4]. A bitewing or a periapical radiograph can be acquired to obtain a high-resolution view centered on the crowns and/or roots of teeth. However, these radiographs have a limited field of view and can be challenging to obtain for certain patients (e.g. small mouth, gag reflex), so that acquiring a PR is envisaged in such cases. While conventional PRs demonstrated limited efficacy in the diagnosis of caries and associated pathologies [5], a potential improvement in their diagnostic accuracy through artificial intelligence (AI) has been suggested [6]. Previous studies have reported on applying convolutional neural networks (CNNs) for the automated segmentation and labeling of dentition on PRs [7–9]. Using transfer learning and transformer-based models, other studies achieved further improvement in the accuracy of these tasks, including the detection and segmentation of teeth and associated numbering [10, 11].

Few studies have performed concurrent tooth detection and classification of caries or periapical lesions on PRs [12–14]. Lower resolution in PRs has been identified as an explanation for their underperformance compared to bitewings and periapical radiographs [15]. Nevertheless, a recently proposed AI method has shown diagnostic capabilities comparable to dentists with 3 to 10 years of experience in diagnosing tooth pathologies while significantly reducing assessment time [6]. These algorithms show considerable potential in improving the detection rates of various pathologies while reducing the work-load associated with radiographic examination [16].

To accelerate the development and benchmarking of AI techniques, several studies have made their PRs and corresponding annotations publicly available in data depositories [13, 17–21]. These public datasets vary in size, from 115 to 4,000 unique PRs, with the annotations ranging from mandible segmentations to the segmentation and labeling of teeth and abnormalities. Despite the availability of these datasets, the adoption of these datasets in dental AI research still needs to be improved. Most studies rely on an in-house dataset, which requires a considerable time investment for annotation. The use of in-house datasets also makes comparisons between studies more difficult, as the annotation guideline to construct each dataset may differ and the local population may be overrepresented.

Therefore, the current study combined two public datasets (OdontoAI [19], DENTEX [21]) to develop an automated method for the novel task of concurrent tooth segmentation, FDI labeling, and diagnosis classification, including caries, impacted tooth, and periapical lesions. This study aimed to evaluate the advantages and challenges of using publicly available datasets in dental AI research, focusing on improving the diagnostic accuracy of caries, impacted teeth, and periapical lesions in PRs.

## Methods

This study was conducted following the code of ethics of the World Medical Association (Declaration of Helsinki) and the checklist of artificial intelligence (AI) in dental research has been consulted for reporting [22]. No informed consent was required as all image data were publicly available and were anonymized.

### Data

A dataset with tooth segmentations (OdontoAI) was pooled with another dataset comprising tooth bounding boxes and associated diagnoses (DENTEX) to develop a two-stage AI method to segment, label, and classify teeth and odontogenic pathologies, such as caries, impacted teeth, and periapical lesions in PRs.

**OdontoAI**: The OdontoAI platform provides a public dataset with 4,000 PRs, of which 2,000 are annotated [19]. The annotations include tooth segmentations with corresponding FDI numbers. The FDI notation describes the location of a tooth by the quadrant in which the tooth resides (1–4) and the numerical order of individual teeth within the quadrant from midline to the back (1–8). PRs were excluded based on the following exclusion criteria: the presence of an artefact (*n* = 1), incorrect or missing tooth segmentation (*n* = 85), edentulous jaws (*n* = 15) or the presence of a mixed dentition (*n* = 238). The remaining 1,661 PRs were split into 1,337 PRs for training and validation and 324 PRs for testing using multi-label stratification based on the FDI numbers [23].

**DENTEX**: The Dental Enumeration and Diagnosis on Panoramic X-rays (DENTEX) challenge provides a public dataset with 705 unique PRs with tooth diagnosis annotations [21, 24]. More specifically, each diagnosed tooth is annotated within a bounding box and labeled as impacted, early caries, deep caries, and/or periapical lesion. Additionally, a subset of 260 PRs includes bounding boxes for teeth without a diagnosis (without pathology). PRs were excluded based on the following exclusion criteria: the presence of an artifact (*n* = 8), incorrect or missing tooth annotations (*n* = 12), or the presence of a mixed dentition (*n* = 3). The remaining 682 PRs were split into 548 PRs for training and validation and 134 PRs for testing using multi-label stratification based on the FDI numbers and tooth diagnoses [23].

### Deep learning method

**Fig. 1:**
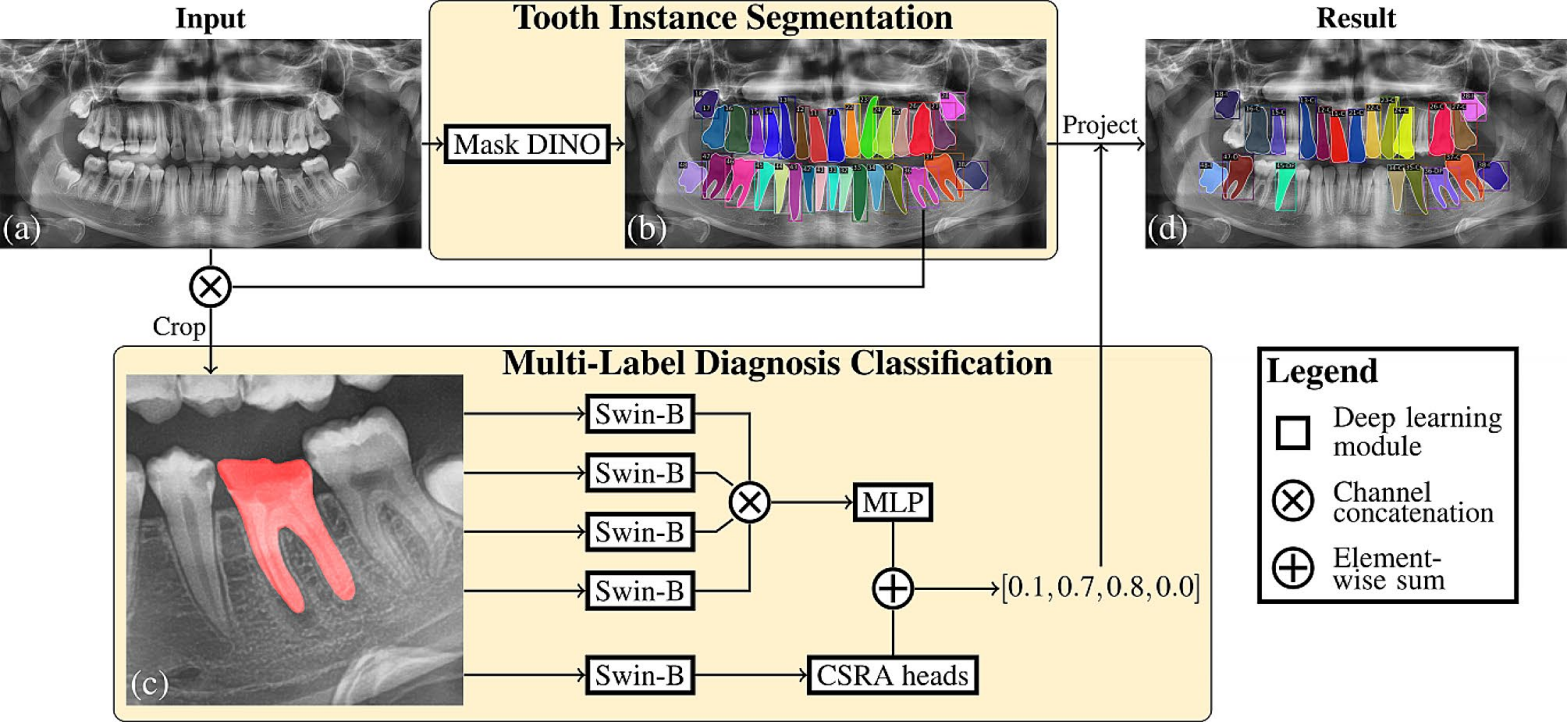
Overview of methodology. The teeth in the input PR **(a)** are segmented by Mask DINO and their FDI numbers are predicted**(b)** [25]. For each predicted tooth, a cropped image is made with the segmentation as extra channel **(c, ROI extraction)**. This image is processed by four binary classifiers, one for each diagnosis, whose predictions are aggregated using an MLP [34], and by a multi-label classifier who returns multiple predictions via its CSRA heads [40]. All multi-label predictions are summed and these final scores are used to add diagnoses to the tooth segmentations **(d)**. Non-diagnosed teeth are predicted, but not shown in the result for clarity. The label is the tooth’s FDI number with C = early caries, D = deep caries, P = periapical lesion, I = impacted

The current method consists of two stages: tooth segmentation and multi-label diagnosis classification. See Fig. 1 for an overview of the method. The first stage for tooth instance segmentation predicted tooth segmentations with corresponding FDI numbers. A region of interest (ROI) was extracted from a PR based on the tooth segmentation; and the second stage for multi-label diagnosis classification predicted multiple abnormalities of the tooth. Training and inference was performed on a workstation with 128GB of system memory and an RTX A6000 GPU with 48GB of memory.

#### Tooth segmentation

Mask DINO was used for tooth segmentation (Fig. 1b). This is a recent end-to-end deep learning pipeline for instance segmentation using vision transformers [25]. For this study, Mask DINO was implemented using the MMDetection framework (v3.1.0) based on PyTorch 2.0.1 [26, 27].

The model was initialized with a ResNet-50 backbone [28] and was pre-trained on the COCO dataset [29]. The pre-trained model was fine-tuned for a maximum of 50 epochs using the AdamW optimizer with a weight decay of 0.05 [30]. The initial learning rate was ${10}^{-4}$ and subsequently adjusted, divided by 10 after epochs 44 and 48. Data augmentation included flipping, resizing, and copy-and-pasting of a tooth on top of the contralateral tooth with the same tooth number [31]. PRs were processed in mini-batches of two. A multi-task loss function was used to supervise the predicted bounding boxes, segmentations, and labels.

For inference, test-time augmentation was applied in the form of flipping, while non-maximum suppression was used to select the final tooth predictions with a set threshold of 0.1 [32]. The minimum bounding box around a tooth segmentation was used as the bounding box for this tooth.

#### Tooth ROI extraction

The fine-tuned Mask DINO model was used to predict all teeth in the PRs from the DENTEX challenge. These tooth predictions were used as inputs for the subsequent diagnosis classification stage.

A predicted tooth bounding box was matched with the reference tooth bounding box with a maximum intersection over union (IoU) of at least 0.25. The diagnosis labels of a predicted tooth were assigned based on the diagnosis labels of the matched reference tooth. Predicted teeth that were not matched were excluded, which resulted in 5,887 non-diagnosed teeth, 593 impacted teeth and 2,110 teeth with early caries, 536 teeth with deep caries, and 150 teeth with a periapical lesion. For each matched tooth, a classification image was generated (Fig. 1c). An additional color channel representing the tooth’s binary segmentation was added to the grayscale PR. This two-channel image was then cropped around the tooth segmentation with a margin of 10%. This margin provided extra contextual information to the classification model, improving its diagnostic effectiveness.

#### Multi-label diagnosis classification

The classification stage was implemented using MMPreTrain (v1.0.1) based on PyTorch 2.0.1 [27, 33]. Classification images extracted for each tooth in the PRs from the DENTEX challenge were used for training and evaluation (Fig. 1c).

##### Pre-training

A Swin-B backbone [34] was first pre-trained on the ImageNet dataset [35] using the SimMIM method [36]. SimMIM is a self-supervised pre-training technique that removes parts of the input image and predicts the missing pixels. This allows it to effectively model the relationships between foreground objects and their context objects. After pre-training on ImageNet for 800 epochs, the Swin-B backbone underwent further pre-training on the train/validation classification images for an additional 100 epochs.

##### Binary classification

Four binary classifiers were trained to distinguish diagnosed teeth from non-diagnosed teeth for each diagnosis. Each classifier comprised a Swin-B backbone followed by a fully-connected layer. The backbone was initialized using the pre-trained model parameters and each classifier was fine-tuned for a maximum of 80 epochs using the AdamW optimizer with a weight decay of 0.05 [30]. The learning rate increased linearly to 0.0002 during the first 5 epochs and subsequently followed a cosine annealing schedule. Data augmentation included flipping, resizing, spatial and intensity transformations [37], and copy-and-pasting a tooth to another classification image with the same FDI number [31]. PRs were processed in mini-batches of 256 and the predictions were supervised using the cross-entropy loss function with label smoothing [38]. Class imbalance was addressed by sampling diagnosed and non-diagnosed teeth equally [39].

##### Multi-label classification

The four binary classifiers were frozen and a multi-layer perceptron (MLP) was used to aggregate their predictions into four diagnostic probabilities. A fifth pre-trained Swin-B backbone was used to learn visual features for multi-label classification. The final feature maps of the Swin-B backbone were further processed by the class-specific residual attention (CSRA) module [40]. This module employed multi-head self-attention to focus on multiple locations of the input image simultaneously. Each head of the CSRA module predicted four diagnostic probabilities. The predictions from the binary classifiers and the predictions from the CSRA module are aggregated by element-wise summing. The same training setup was used as in the binary classification stage, with the exception that class imbalance was addressed by more frequent sampling of classification images with rare diagnoses [41]. Furthermore, the focal loss function was used [42].

##### Inference

During the inference stage, test-time augmentation was applied with flipping, and the predictions were aggregated by averaging the diagnostic probabilities. The probabilities were updated to incorporate prior knowledge. More specifically, each diagnostic probability was multiplied by the score of the tooth segmentation. Mutual exclusions were downscaled between early caries and deep caries, as well as between impacted teeth and other diagnoses, and between impacted teeth and teeth other than third molars. Finally, the predicted diagnoses of the extracted ROI were projected back to the same tooth of the input PR (Fig. 1d).

### Evaluation and comparison

Five-fold cross-validation splits were determined for the train/validation PRs from OdontoAI and DENTEX datasets using multi-label stratification [23]. Both stages are trained five times given a different cross-validation split to investigate the variability of the method’s results. Both the tooth segmentation and diagnosis classification stages were trained five times with different cross- validation splits to investigate the variability of the method’s results.

The performance of the tooth segmentation stage was evaluated using a free-response receiver-operating-characteristics (FROC) analysis and mean average precision (mAP) metrics. These analyses and metrics were calculated as the mean performance on the test split that was held out for each round of cross-validation.

The diagnosis classification stage was evaluated based on a ROC analysis for each type of diagnosis. Additional metrics such as accuracy, F1-score, and AUC were also computed and reported. To gain insights into the model’s limitations, failure cases were shown and analyzed to assess the method’s errors.

### Statistical analysis

The model predictions on the test splits were compared to the reference annotations using scikit-learn (v1.3.0). Classification metrics were reported as follows: accuracy = $\frac{TP+TN}{TP+TN+FP+FN}$ and F1-score = $\frac{2TP}{2TP+FP+FN}$, where TP, TN, FP, and FN denote true positives, true negatives, false positives, and false negatives, respectively. Furthermore, the area under the receiver-operating-characteristics curve (AUC) and the confusion matrix were presented.

### Results

**Fig. 2:**
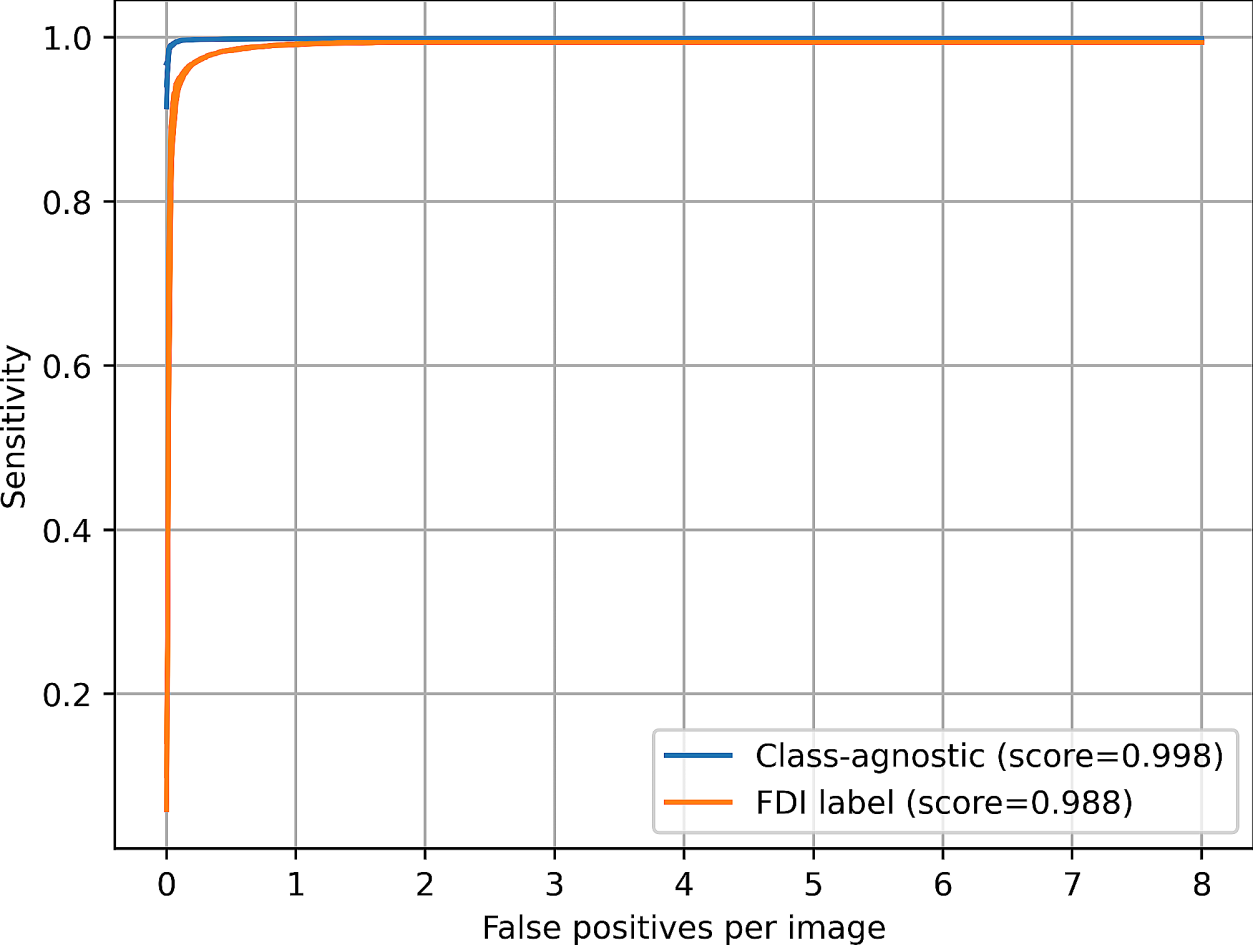
Free-response receiver operating characteristic (FROC) curves of tooth instance segmentation on PRs. Results are based on 324 held-out test PRs from the OdontoAI platform. The score is computed as the mean sensitivity at false-positive rates of 0.25, 0.5, 1, 2, 4, and 8 following [48]

The current method demonstrated high performance in automated tooth segmentation and labeling with a mean FROC score of 0.988 and a mAP of 0.848 (Fig. 2; Table 1).

**Table 1:** Tooth instance segmentation metrics.The results on the held-out test split of PRs from the OdontoAI platform are averaged for five models trained with 5-fold cross-validation. mAP@IoU=$x$ denotes mean average precision at intersection over union threshold(s) $x$

mAP@IoU =	0.5	0.75	0.5:0.95
Class-agnostic	0.990	**0.989**	**0.849**
FDI	**0.993**	0.985	0.848

**Fig. 3:**
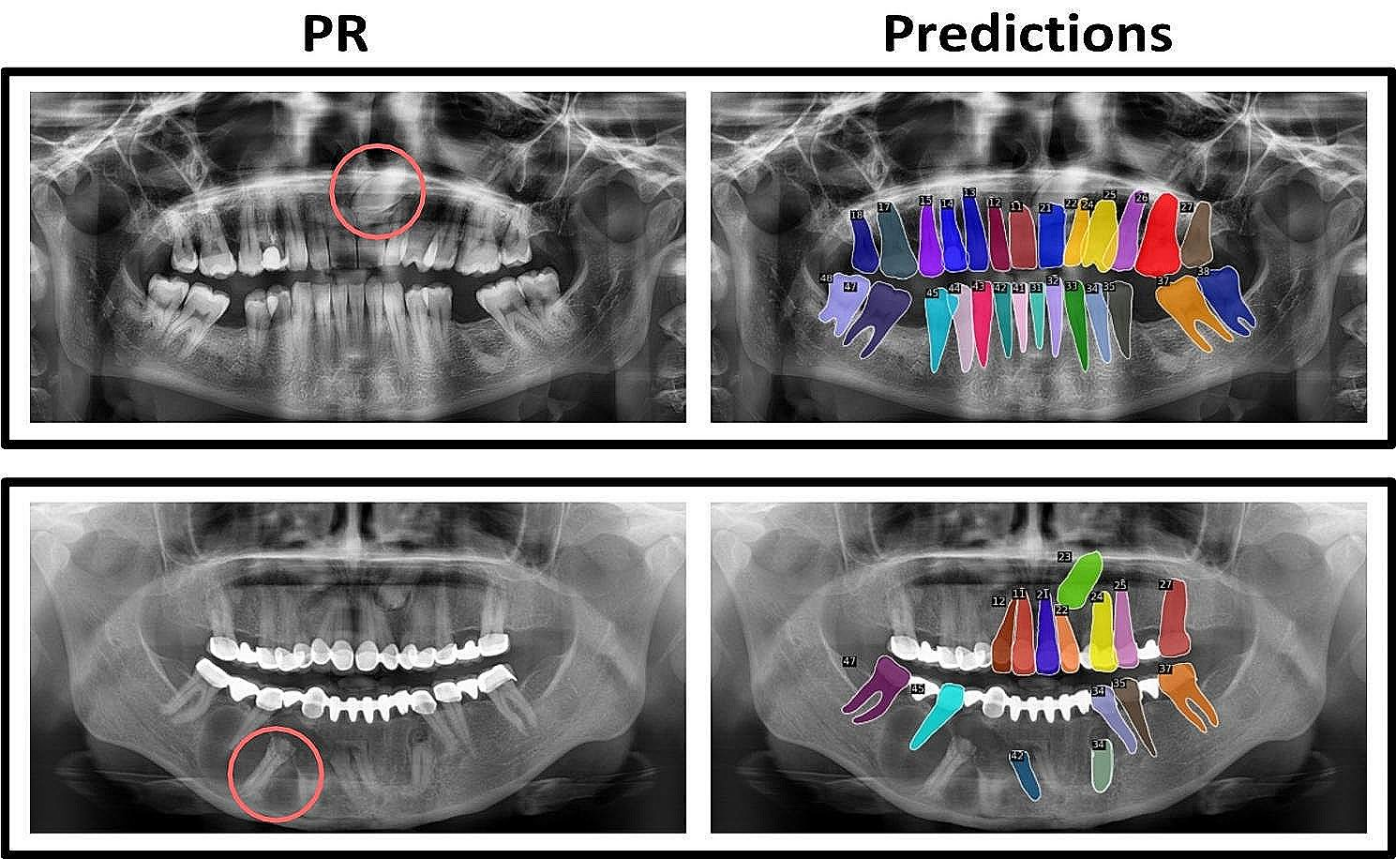
Tooth segmentation failure cases. Two PRs from the DENTEX challenge dataset are shown with predictions. A horizontally impacted canine (in red circle) is not segmented in the first PR and multiple teeth (e.g. in red circle) are not segmented in the second PR due to a syndromic disease. The label is the tooth’s FDI number

When the FDI numbers were excluded from consideration, the performance increased further, with a mean FROC score of 0.998 and a mAP of 0.849. Upon visual examination, the tooth segmentations were accurate, even in cases of overlapping teeth (Fig. 1). Errors that were present could be attributed to poor image quality or uncommon dental anomalies, such as horizontally impacted canines or the presence of a syndromic disease (Fig. 3).

**Fig. 4:**
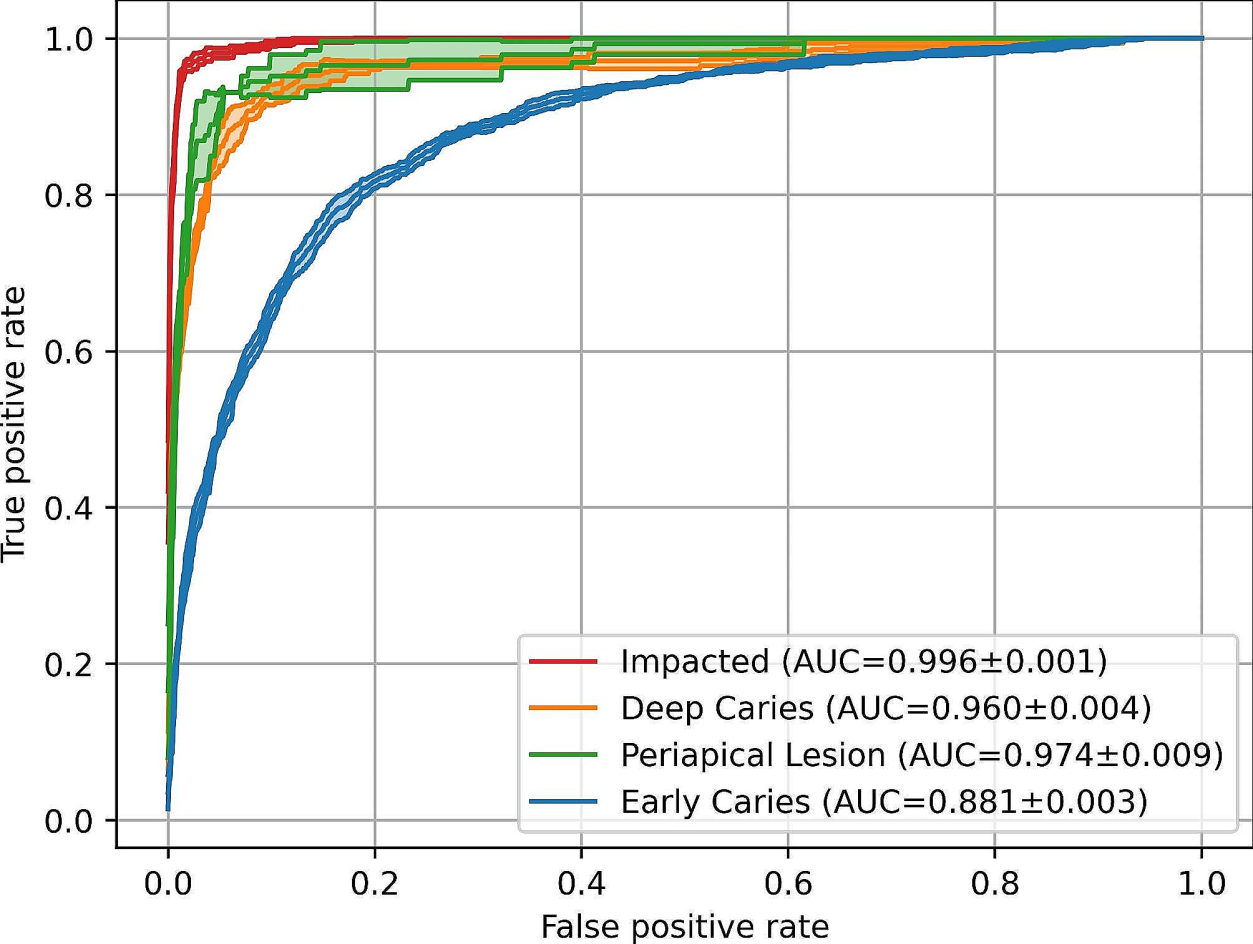
Receiver operating characteristic (ROC) curves illustrating the multi-label classification results of tooth diagnoses on PRs. A varying effectiveness can be observed for different tooth diagnoses. Results are based on 134 held-out test PRs from the DENTEX challenge. AUC = area under ROC curve

The current method also achieved moderate to high accuracies in classifying dental pathology (Fig. 4). The method was most effective in classifying impacted teeth (AUC = 0.996), followed by teeth with a periapical lesion (AUC = 0.974) and/or a deep caries lesion (AUC = 0.960), and the model was least effective in detection of teeth with an early caries lesion (AUC = 0.881).

**Fig. 5:**
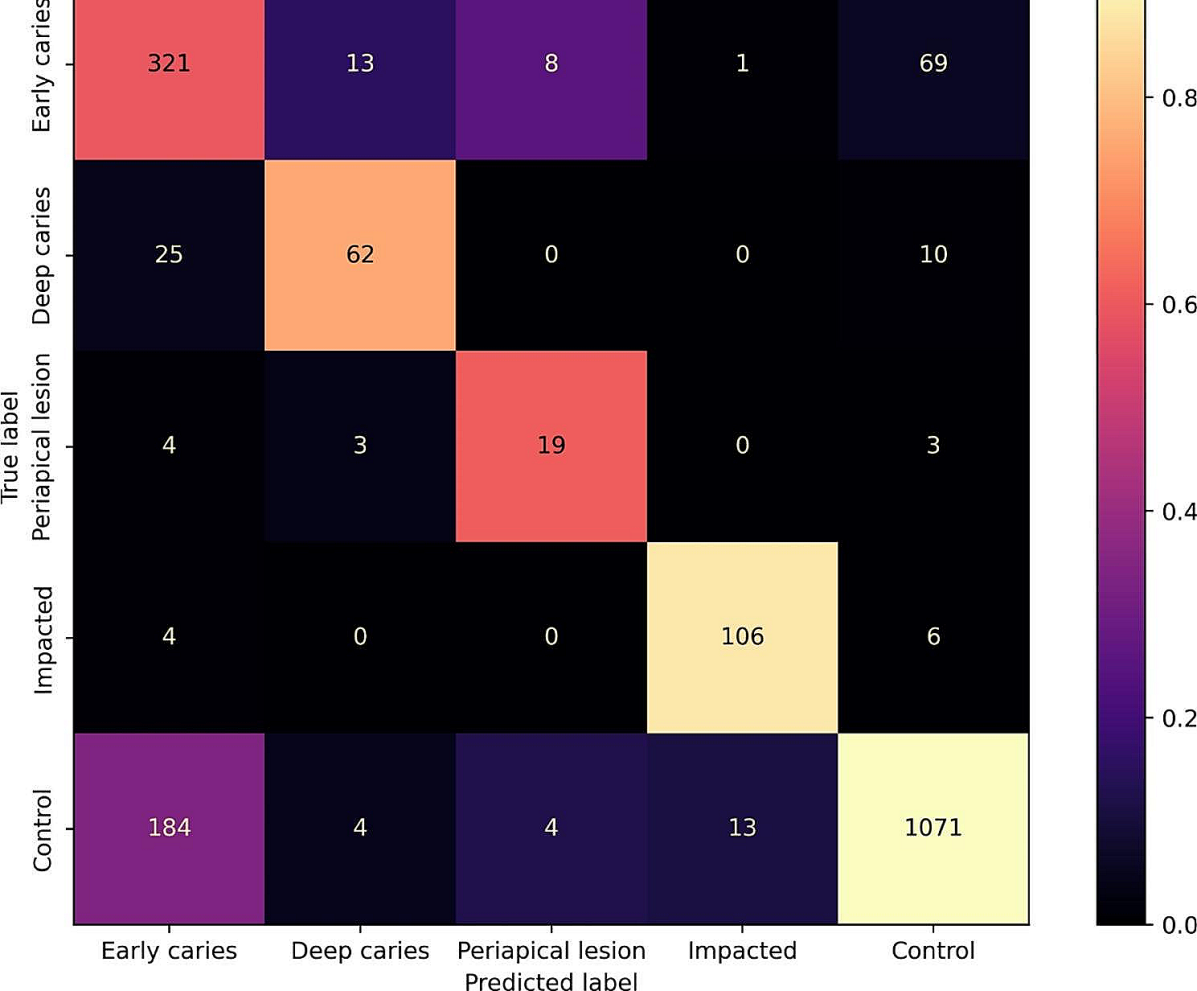
Confusion matrix illustrating the multi-label classification results of tooth diagnoses on PRs. Results on 134 held-out test PRs from the DENTEX challenge are shown for the most effective model. The colorbar is normalized according to the number of PRs per predicted label

The present two-stage AI model had difficulties in distinguishing between teeth with early caries and non-diagnosed teeth, as shown in Fig. 5. In cases where the reference or the predictions indicated a diagnosis (excluding the true negatives, see Table 2), classifying impacted teeth yielded the highest effectiveness (F1 = 0.901), followed by deep caries (F1 = 0.683), early caries (F1 = 0.662), and periapical lesions (F1 = 0.603).

**Table 2:** Multi-label tooth diagnosis classification metrics. The results on the held-out test split are averaged for five models trained with 5-fold cross-validation. See subsection 3.4 for elaboration on the metrics

	Accuracy	F1-score	AUC
Early Caries	0.843	0.662	0.881
Deep Caries	0.969	0.683	0.960
Periapical Lesion	0.987	0.603	0.974
Impacted	0.988	0.901	0.996

## Discussion

This study aimed to explore the advantages and challenges of employing publicly available research data for AI-based dental research. Based on two public datasets, OdontoAI and DENTEX, a two-stage AI model was developed for automated tooth segmentation and diagnosis classification in PRs, to detect impacted teeth as well as teeth with (deep) caries and/or periapical lesions. Using the Mask DINO model (vision transformer), segmentation and labeling of teeth was accurate, obtaining a mAP of 0.848. The AI model demonstrated high effectiveness in the multi-label diagnosis classification of impacted teeth (F1-score = 0.901; AUC = 0.996). However, it showed limitations in detecting teeth with early caries (F1-score = 0.662; AUC = 0.881).

The present study highlighted the potential benefits of utilizing public research datasets to develop AI-based approaches to perform specific tasks, to aid clinicians in daily practice. By combining two distinct datasets, data collection and data annotation time could be considerably reduced, yet time was required for adequate data selection. Furthermore, the two-stage AI model was trained to use reference annotations from each dataset effectively. This demonstrated that using and combining public research datasets is a viable way to develop innovative dental AI solutions.

Many studies have been performed investigating tooth segmentation in PRs [43]. A recent study annotated 6,046 PRs and developed a two-stage model that first segmented and cropped around a region of interest, whereafter tooth segmentations were predicted and labeled with FDI numbers [44]. The authors reported an mAP of 0.966 at an intersection over union (IoU) threshold of 0.75 on the validation set, which was less effective compared to our method. Another study used a dataset comprising 1,500 PRs and incorporated individual models for tooth segmentation and tooth labeling using collaborative learning [7]. The results showed an mAP of 0.973 at an IoU threshold of 0.5, which was less effective compared to our model. The high effectiveness of the current method could be partly explained by the curation of the dataset from the OdontoAI platform; dental implants and bridges were not annotated and were not included for model evaluation [19].

One study investigated the efficacy of automatic software for classifying dental conditions such as restorations, caries, and periapical lesions [45]. This software achieved F1-scores of 0.593 and 0.479 for classifying teeth with caries and periapical lesions, respectively. In contrast, the present study achieved higher F1-scores of 0.662 and 0.603 for these conditions. Another study used a two-stage approach similar to the present study, using manually segmented and cropped third molars in PRs to determine the presence of caries lesions [46]. The study reported an F1-score of 0.86 and an AUC of 0.90, showing better results compared to the current study. However, this AI method has limited utility compared to the current method, as it requires clinician interactions and only assesses caries in third molars. The highlighted studies made use of datasets without public access and the source code of their methods was unavailable, making a thorough reproduction unfeasible. Direct comparisons between the results of the current method and the highlighted studies should thus be made with caution.

Several studies have focused on predicting the segmentation of caries and periapical lesions on PRs. CariesNet was trained on 1159 PRs to predict a segmentation of shallow, moderate, and deep caries lesions and achieved a Dice similarity coefficient (DSC) of 0.935 [12]. A second study used a U-net model to segment periapical lesions in 470 PRs and detected the lesions with an F1-score of 0.88 at an IoU threshold of 0.70 [47]. These studies suggest that a direct segmentation of caries and periapical lesions provides a more precise reference annotation that can be used to develop more effective AI methods. However, acquiring reference segmentations is considerably more laborious, time-consuming, and resource-intensive than identifying or labeling tooth diagnoses. Achieving a consensus among dental experts on the exact boundaries and nature of the segmented lesions poses an additional challenge. This makes it more difficult to establish a unanimous reference for training AI models.

**Fig. 6:**
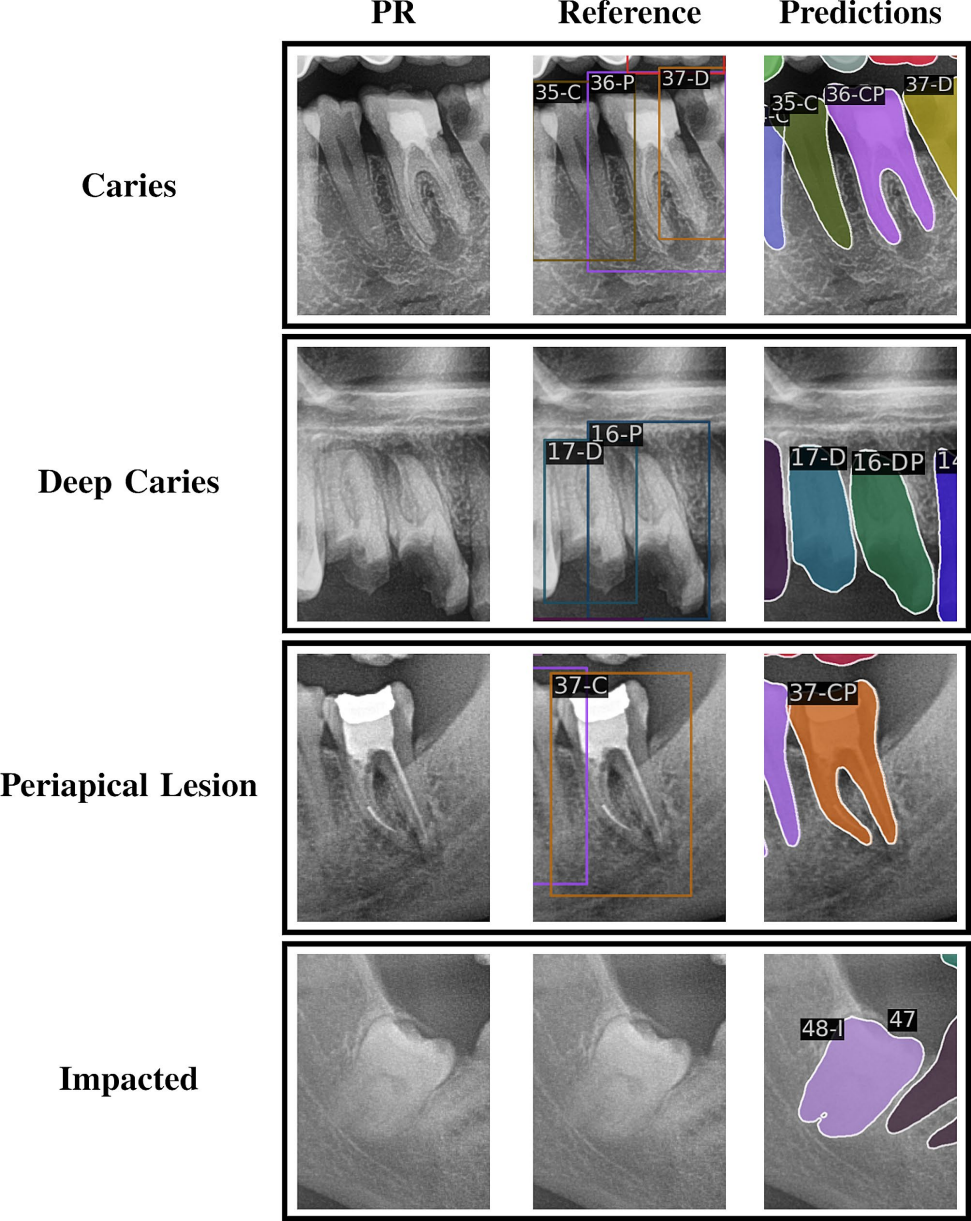
DENTEX annotations with missing diagnoses. A missed diagnosis (false negative) is shown for each diagnosis, which are selected based on the maximum difference between a diagnosis probability and whether that diagnosis is annotated. Note that the DENTEX dataset only annotates diagnosed teeth, whereas the current method predicts all teeth. The label is the tooth’s FDI number with C = early caries, D = deep caries, P = periapical lesion, I = impacted

**Fig. 7:**
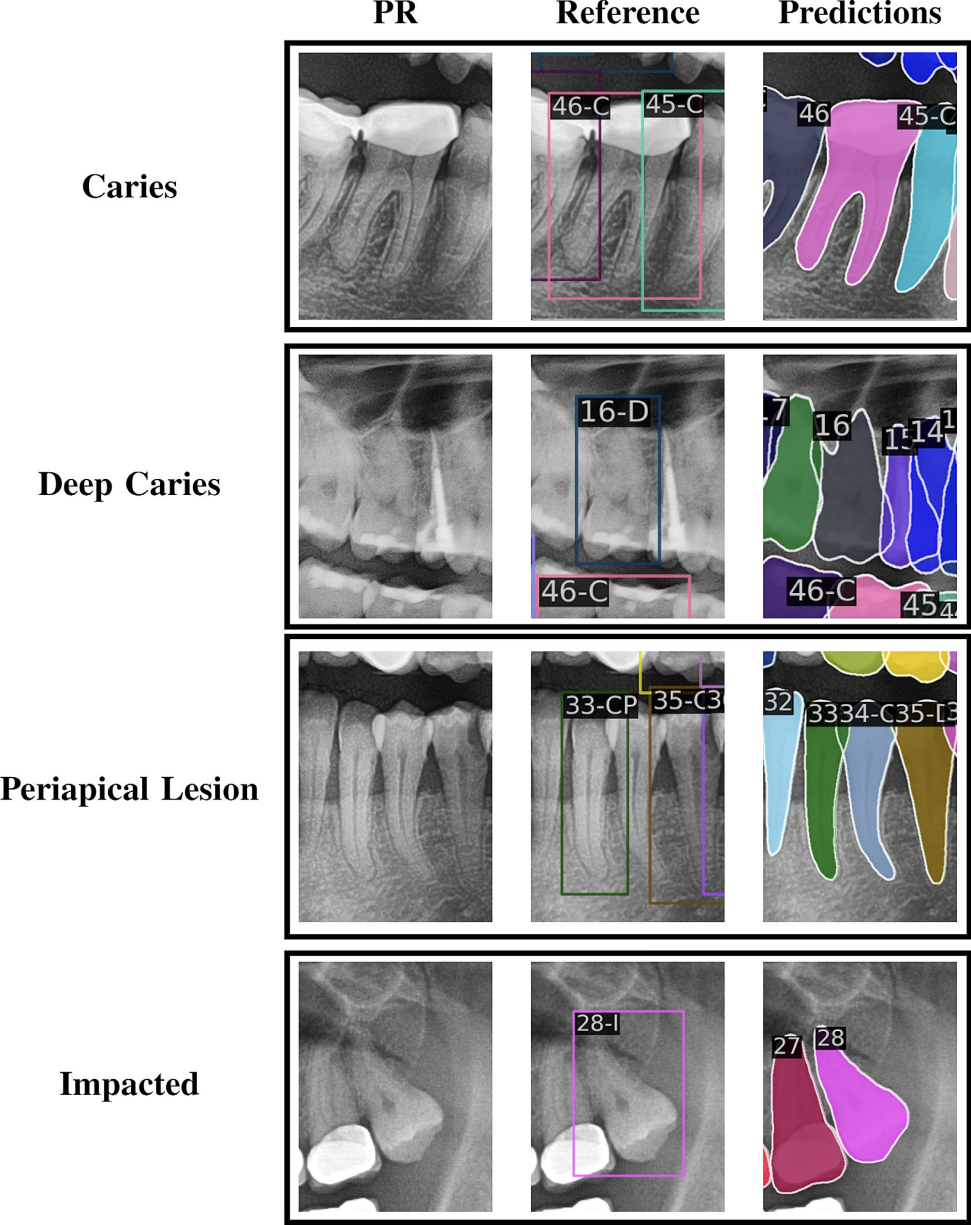
Misdiagnosed DENTEX annotations. A misdiagnosis (false positive) is shown for each diagnosis, which are selected based on the maximum difference between a diagnosis probability and whether that diagnosis is annotated. Note that the DENTEX dataset only annotates diagnosed teeth, whereas the current method predicts all teeth. The label is the tooth’s FDI number with C = early caries, D = deep caries, P = periapical lesion, I = impacted

A limitation of this study is the inconsistency of the dataset with tooth diagnosis annotations, as shown in Figs. 6 and 7. As the tooth bounding box can be notably larger than the assessed tooth, some reference teeth with diagnoses could not be matched to a predicted tooth during the construction of classification images (subsubsection 3.2.2).

Moreover, the dataset contained missing diagnoses (false negatives) and misdiagnoses (false positives). As a result, a re-assessment of the dataset could be performed based on the largest discrepancies between the reference annotations and the AI method’s predictions to improve the consistency of the dataset and the model’s effectiveness. Biases present in the dataset and model could only be identified and corrected with a diagnostic re-assessment of (a sample of) the dataset involving a dental expert. Another limitation is the long processing time of the current two-stage model (40 s), compared to single-stage object detectors or segmentation models. This could potentially hinder the adoption of the AI method, as a clinician expects immediate results upon acquiring a PR.

The current work can be extended by incorporating additional public research data with segmentations of caries and periapical lesions [13, 20]. Using the current method for tooth segmentation, it is possible to integrate lesion segmentations to form multi-level tooth segmentations, with the tooth segmentation as the first level and the associated lesion segmentations as the second level. However, performing a diagnostic re-assessment to verify and validate these datasets before using them for further research is recommended. Another direction for future work is collecting PRs and bitewings or periapical radiographs from the same patient visit. Clinicians make fewer diagnostic errors when detecting caries and periapical lesions on these higher-resolution radiographs. Subsequently, a dataset of PRs with more reliable annotations of tooth diagnoses can be created by making the diagnoses on the associated higher-resolution radiographs.

### Conclusions

This study aimed to investigate the opportunities and challenges of using publicly available datasets in dental AI research. For that purpose, two public datasets with panoramic radiographs were combined to develop an effective method for predicting tooth segmentations, FDI numbers, and tooth diagnoses, concurrently. Using and combining public datasets for AI research in dentistry enables fast exploration of novel tasks and considerably reduces the development time of AI methods. However, the quality of the reference annotations can vary greatly depending on the dataset. Implementing a form of data quality assurance is therefore recommended to optimize the performance of AI models while limiting the risk of biases.

## Data Availability

The DENTEX challenge dataset used during the current study is available in the Zenodo repository, https://zenodo.org/record/7812323#.ZDQE1uxBwUG, and the dataset from the OdontoAI platform can be downloaded from the platform’s website, https://odontoai.com/dataset/.

## Abbreviations

AI: Artificial Intelligence
AUC: area under receiver operating characteristic curve CNNs = convolutional neural networks
COCO: Common Objects in Context CSRA = class-specific residual attention
DENTEX: Dental Enumeration and Diagnosis on Panoramic X-rays
DINO: DIstillation of knowledge with NO labels
DSC: Dice similarity coefficient
FDI: Fédération Dentaire Internationale
FROC: free-response receiver operating characteristic IoU = intersection over union
mAP: mean average precision
PRs: Panoramic radiographs
ROC: receiver operating characteristic
SimMIM: simple masked image modeling
